# Osteonecrosis of femoral head in young patients with femoral neck fracture: a retrospective study of 250 patients followed for average of 7.5 years

**DOI:** 10.1186/s13018-020-01724-4

**Published:** 2020-06-29

**Authors:** Fang Pei, Rui Zhao, Fenglei Li, Xiangyang Chen, KaiJin Guo, Liang Zhu

**Affiliations:** 1 Department of Orthopedics, Nanjing Medical University, Nanjing, Jiangsu P.R. China; 2 Department of Orthopedics, The Affiliated Hospital of Xuzhou Medical University, Xuzhou, Jiangsu China; 3 Department of Radiology, Xuzhou Municipal Hospital Affiliated of Xuzhou Medical University, Xuzhou, Jiangsu China; 4 Department of Orthopedics, Xuzhou Central Hospital, Xuzhou, Jiangsu China

**Keywords:** Femoral neck fracture, Osteonecrosis of the femoral head, Risk factors, Internal fixation

## Abstract

**Objective:**

To investigate the risk factors for osteonecrosis of the femoral head (ONFH) after the treatment of femoral neck fracture in patients under 60 years old.

**Methods:**

A total of 250 cases of femoral neck fracture treated at 3 hospitals in Xuzhou from January 2002 to January 2016 were studied. The patients were followed up for 1~15 years, and the clinical data on femoral head necrosis after the femoral neck operation were analysed retrospectively. Risk factors were recorded, including age, gender, preoperative traction, time from injury to operation, reduction method, type of reduction, BMI, ASA classification, and quality of reduction. Logistic regression analysis was used to evaluate the independent risk factors for ONFH after treatment of femoral neck fracture.

**Results:**

The duration of follow-up was 1~15 years, with an average of 7.5 years. None of the 250 patients had fracture non-union, but 40 (16%) had necrosis of the femoral head. The time to necrosis of the femoral head was 1~7 years after the operation, with an average of 3.8 years. Univariate analysis showed that the type of fracture, the quality of reduction, the removal of internal fixation, BMI and ASA classification were risk factors affecting necrosis of the femoral head in patients with femoral neck fracture, and the difference was statistically significant (*P* < 0.05). Multivariate analysis showed that internal fixation, fracture type (displacement), reduction quality (dissatisfaction), BMI (> 25), and ASA grade (III + IV) were independent risk factors affecting femoral head necrosis in patients with femoral neck fracture.

**Conclusion:**

A variety of high-risk factors for femoral head necrosis are present after surgery with hollow compression screws for femoral neck fracture in adults. Removal of internal fixation, type of fracture, quality of reduction, BMI, and ASA classification were the most important risk factors influencing the development of femoral head necrosis. During treatment, there should be some targeted measures to reduce the incidence of necrosis of the femoral head.

## Introduction

With the gradual aging of China’s population, the proportion of elderly diseases in the overall disease spectrum is increasing. Worldwide, the incidence of femoral neck fractures is increasing annually [1–3]. In 1990, the total number of hip fractures in the world was 1,000,660, and it is expected to reach 6,000,260 by 2050 [4, 5]. Femoral neck fractures have become a growing burden on global health care expenditure. There is a special blood supply system in the neck of the femur, and a series of complications, such as necrosis of the femoral head, can be caused by improper treatment after the fracture, which brings great pain to the patients. Osteonecrosis of the femoral head (ONFH) is a rare but disastrous complication. Internal fixation of femoral neck fractures has been proven to be a safe and effective treatment [6]. However, because of the special anatomical structure and blood supply characteristics of the femoral neck and femoral head, the necrosis rate of the femoral head after operation is 10–30% [7, 8]. The majority of the closed reduction and internal fixation procedures are performed in young and middle-aged patients under the age of 60, and Chinese doctors are more inclined to perform artificial hip replacement when the patient is over 60 years old [9]. Femoral neck fracture in patients under 60 years of age is related to trauma of the femoral neck and has a high incidence of necrosis of the femoral head, which will seriously affect quality of life [10]. The younger the patient is, the greater the need for the operation to recover original hip function; thus, doctors in the Department of Orthopedics require knowledge on femoral head necrosis after the femoral neck fracture. The related risk factors can minimize the occurrence of risk factors and improve the prognosis of patients. With medical advances, patients with femoral neck fractures are increasingly inclined to undergo surgical treatment [11]. However, there is still much controversial over the treatment of femoral neck fractures in patients under 60 years of age.

## Patient data and methods

### Patient data

The clinical data of patients with femoral neck fracture treated by internal fixation in three hospitals from January 2002 to January 2016 were retrospectively analyzed. This retrospective study was approved by our institutional review board. Inclusion criteria are as follows: (1) age ranging from 18 to 60 years; (2) treatment with hollow compression screws; (3) unilateral femoral neck fracture; and (3) informed consent. Exclusion criteria are as follows: (1) pathological fracture and (2) incomplete clinical data or follow-up data. Finally, 250 patients were included in the analysis, including 113 males and 137 females aged 18~59 years, with an average age of 56.4 ± 6.8 years. The causes of injury included 106 cases of traffic injuries, 79 cases of falling injuries, and 65 cases of falls.

### Surgical methods

All patients were operated under general anaesthesia while lying on the traction bed; the patients underwent closed reduction first, and then, X-ray was used to confirm the effect of reduction. When the closed reduction was satisfactory, 2~3 needles were inserted in the femoral head from 3 to 5 cm below the greater trochanter; the fracture and screw positions were observed by X-ray, and 2~3 hollow screws were affixed along the guide needle. If the closed reduction failed, open reduction and internal fixation were performed. In open reduction and internal fixation procedure, an anterolateral incision of approximately 12 cm in length was made; the incision was made layer by layer, and the space between the tensor fascia tensor muscle and the gluteus maximus was separated. The medial gluteal and lateral femoral muscles were exposed, the middle gluteal 1/3 was cut, the fascia of the gluteus medius muscle and the lateral femoral muscle fascia were maintained, the joint capsule was exposed, the joint sac was opened and cleared, the intra-articular haematoma was cleared, and the fracture was reduced. Following a satisfactory reduction, 2~3 needles were inserted in the femoral head from 3~5 cm below the greater trochanter. The fracture and screw positions were observed by X-ray and 2~3 hollow screws were affixed along the guide needle. The incision was then rinsed and stitched layer by layer. One drainage tube was placed in the incision.

### Clinical evaluation

(1) Clinical data were collected as follows: a basic self-designed questionnaire was used to collect basic data. The questionnaire included factors affecting femoral head necrosis, such as age, gender, displacement of fractures, medical complications, and postoperative weight-bearing. In addition, time, preoperative traction, removal of internal fixation, fracture side, Harris hip score, etc., were noted (2) The assessment criteria for internal medicine comorbidities were based on those of the American Society of Anaesthesiologists (ASA): level I: healthy; level II: mild systemic disease; level III: combined severe, uncontrollable systemic disease; level IV: uncontrolled and life-threatening systemic disease; and level V: within 24 h, patients undergoing surgery may die. (3) The stage of femoral head necrosis was determined using the Ficat classification criteria: stage 0: the patient has no clinical symptoms, and X-ray findings are normal; stage I: X-ray findings are normal, or there is mild osteoporosis, limited activity, or hip pain; stage II: limited pain and mobility in the hip of the patient, and X-ray films show diffuse bone hardening and normal femoral head morphology; stage III: X-ray films show a collapse of the femoral head greater than 2 cm and a normal joint space, and the patient experiences hip pain and displays limited activity; and Stage IV: In the period of osteoarthritis, the X-ray shows collapse of the femoral head, a narrow joint space, and loss of articular cartilage, and the patient experiences joint pain and exhibits varying activities of the joint.

### Follow-up

After discharge, the patient was asked to go to the clinic once a month until the fracture healed or necrosis of the femoral head occurred. After the fracture healed, the patient was rechecked once a year until necrosis of the femoral head or death occurred. If the patient could not come to the hospital for re-examination, the patient was required to undergo X-ray imaging in the local hospital and then pass the images to the researchers through Email or post and express mail.

### Statistical analysis

SPSS 20 statistical software was used for statistical analysis. Single-variable analysis was performed by the chi-square test, and multivariate analysis was performed by logistic regression analysis. *P* < 0.05 indicated statistical significance.

## Results

### Follow-up results

**Fig. 1:**
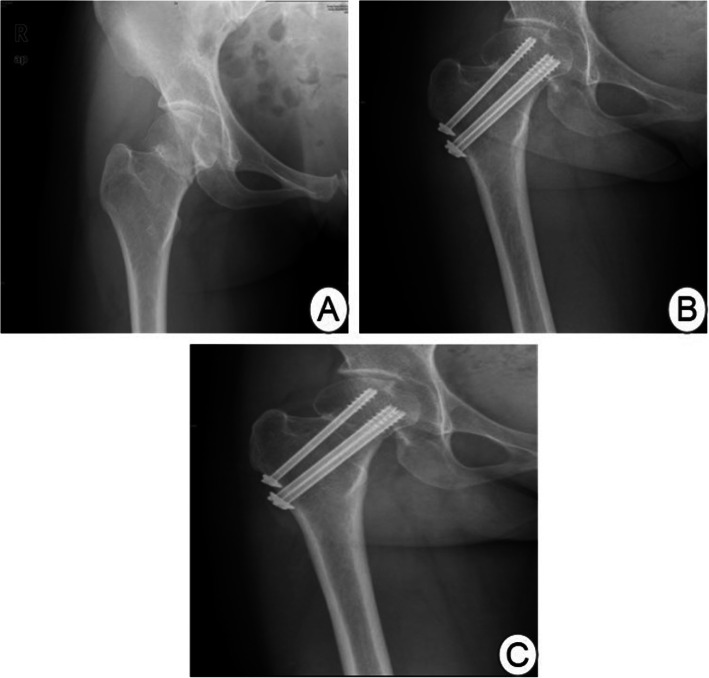
**a** Preoperative X-ray film. **b** At 1 year after the operation, X-ray examination showed that the necrosis of the right femoral head. **c** At 2 years after the operation

The duration of follow-up was 1~15 years, with an average of 7.5 years. None of the 250 patients had fracture non-union, but 40 (16%) had necrosis of the femoral head. The time to necrosis of the femoral head was 1~7 years after the operation, with an average of 3.8 years. Figure 1 shows a typical case of a male patient aged 36 years old who suffered from a right femoral neck fracture caused by a car accident. One year after the operation, X-ray examination showed necrosis of the right femoral head (Fig. 1). In the No-ONFH group, the Harris hip score of 139 patients was more than 80 scores, and the satisfaction rate was as high as 66.2% (139/210). In the ONFH group, only 7 patients’ HSS score were more than 80 scores, and their satisfaction rate was only 17.5% (7/40).

### Univariate analysis of factors affecting the occurrence of ONFH

The results of univariate analysis showed that the type of fracture (*χ*^*2*^ = 8.316, *P* = 0.004), the quality of reduction (*χ*^*2*^ = 14.345, *P* < 0.001), the removal of internal fixation (*χ*^*2*^ = 7.459, *P* = 0.006), BMI (*χ*^*2*^ = 17.769, *P* < 0.001), and ASA (*χ*^*2*^ = 13.462, *P* < 0.001) classification were risk factors affecting ONFH in patients with femoral neck fracture, and the difference was statistically significant (Table 1). There were no statistically significant differences in the ONFH between patients who underwent operations before or after the 24 h interval (*P* < 0.05) (Table 2). Similarly, age, gender, fracture season and reduction method did not represent a predictive factor for complications in the univariate logistic regression analysis (*P* > 0.05).

**Table 1:** Univariate analysis of factors affecting femoral head necrosis

Index	n	ONFH group (*n* = 40)	No-ONFH group (*n* = 210)	*χ*^*2*^	*P*
Age				0.940	0.625
18~25	25	5	20		
26~40	35	7	28		
41~60	190	28	162		
Gender				0.001	0.978
Male	113	18	95		
Female	137	22	115		
Preoperative traction				2.268	0.132
Yes	83	24	151		
No	167	16	59		
Time from injury to operation (h)				0.021	0.884
≤ 24	85	14	71		
≥ 24	165	26	139		
Reduction method				0.005	0.941
Closed reduction	80	13	67		
Opened reduction	170	27	143		
Start time of postoperative weight-bearing (months)				0.733	0.693
< 3	23	5	18		
3~6	197	31	166		
> 6	30	4	26		
Type of fracture				8.316	**0.004**
No-displacement fracture	108	9	99		
Displacement fracture	142	31	111		
Quality of reduction				14.345	**< 0.001**
Satisfied	165	16	149		
Dissatisfied	85	24	61		
Removal of internal fixation				7.459	**0.006**
Yes	171	20	151		
No	79	20	59		
BMI (kg/m^2^)				17.769	**< 0.001**
≥ 25	79	24	55		
< 25	171	16	155		
Fracture season				0.082	0.994
Spring	53	8	45		
Summer	41	7	34		
Autumn	36	6	30		
Winter	120	19	101		
ASA classification				13.462	**< 0.001**
I + II	135	11	124		
I II + IV	115	29	86		
Harris Hip score					
> 90	20	0	20		
81–90	126	7	119		
71–80	80	15	65		
< 70	24	18	6		

Harris Hip score: *> 90* very good, *81–90* good, *71–80* neutral, *< 70* not good

**Table 2:** Multivariate analysis of factors affecting the occurrence of ONHF

Index	ß	SE	Wald	*P*	OR	95%CI
Removal of internal fixation	0.372	0.175	4.871	**0.028**	1.647	1.105~2.547
Type of fracture (displacement fracture)	0.325	0.135	6.254	**0.012**	1.398	1.071~1.996
Quality of reduction (dissatisfied)	0.492	0.243	3.968	**0.031**	1.625	1.008~2.658
BMI (≥ 25)	1.368	0.389	11.327	**0.019**	4.658	3.658~8.954
ASAS classification (III + IV)	0.462	0.201	2.354	**0.037**	1.697	1.065~2.634

### Multivariate analysis of factors affecting the occurrence of ONFH

All meaningful indexes in the univariate analysis were included in the multi-factor model. The results showed that removal of internal fixation (OR = 1.647, *P* = 0.028), fracture type (displacement) (OR = 1.398, *P* = 0.012), reduction quality (dissatisfaction) (OR = 1.625, *P* = 0.031), BMI (> 25) (OR = 4.658, *P* = 0.019), and ASA grade (III + IV) (OR = 1.697, *P* = 0.037) were independent risk factors affecting femoral head necrosis in the patients with femoral neck fracture (Table 2). Calculated odds ratios (OR) for differences in BMI were 4.658 (95% CI 3.658~8.954). Therefore, BMI (> 25) were 4.658 times more likely to develop ONFH than BMI (< 25). For fracture classification, the incidence of ONFH in displaced fractures was 1.4 times that of non-displaced fractures.

## Discussion

Open reduction and internal fixation surgery is relatively simple, with low surgical trauma and bleeding. Some studies have reported that patients with femoral neck fractures should be treated with open reduction and internal fixation as early as possible to reduce the incidence of non-union and femoral head necrosis [12–14]. However, some scholars hold different views. Raaymakers et al. [15] believed that there is no significant correlation between delayed surgery and surgical outcome in femoral neck fractures. Araujo’s study founded that there was no significant difference in the incidence of femoral head necrosis between early and late surgery for patients with femoral neck fractures but showed that early surgery can significantly reduce pain in the patients [16]. At present, the commonly used internal fixation methods include hollow screw internal fixation, sliding screws, and lateral steel plate internal fixation. However, regardless of the internal fixation method, a good reset is necessary. Hollow screw fixation involves the insertion of three hollow compression screws into the femoral neck with an isosceles triangle position, and its shear and torsion resistance is better than that of other methods. The hollow screw fixation procedure is suitable for fractures with small displacement and no significant degeneration of joints. The aim is to preserve the patient's hip joint as much as possible [11]. Hollow screw internal fixation can be performed percutaneously, with low surgical trauma, little damage to the femoral head blood flow, and preservation of the femoral head; hence, this method is becoming the mainstream choice for internal fixation. Although there are many advantages in open reduction and internal fixation, there are still some defects, such as long fracture healing time, the need to prohibit weight-bearing after operation, possible necrosis of the femoral head, non-union of the bone, and the need for secondary surgery. Secondary surgery not only increases economic costs but also increases trauma to the patient. Elderly patients with femoral neck fractures are generally in poor condition and exhibit insufficient endurance. Therefore, open reduction and internal fixation should be carefully considered as the surgical treatment for femoral neck fractures in the elderly [17].

For young patients, some factors are considered to be closely related to postoperative femoral head necrosis. However, it is still unclear which are the key factors affecting femoral head necrosis [18]. The results of this study indicate that internal fixation removal, fracture type, reduction quality, BMI, and ASA grading are the five key factors affecting internal femoral head necrosis after internal fixation in the treatment of femoral neck fractures.

In this group of patients, the incidence of ONFH after a non-displaced fracture was 8.3% (9/108), which was significantly lower than the postoperative incidence of displaced fractures (21.8%, 31/142). Previous studies have also shown that compared with non-displaced (grade I or II) femoral neck fractures, displaced fractures have a significantly higher incidence of femoral head necrosis after fixation (5 to 20%: 20 to 44%) [2, 19]. Wang et al. [2] analyzed 146 patients with femoral neck fractures and founded that the rate of femoral head necrosis was 20% for displaced fractures and only 5.4% for non-displaced fractures. The incidence of femoral head necrosis is mainly due to fracture displacement, which indirectly indicated that there is of great violence during fracture. Damages to the main blood supply of the femoral head (deep branch of the medial femoral artery and its branches) results in blood flow disruption, reduced femoral head blood flow, decreased intramedullary oxygen partial pressure, decreased sub-choroidal blood flow and oxygen partial pressure, bone cell ischemia and hypoxia and, finally, femoral head necrosis. In addition, fracture displacement also increases the difficulty of fracture reduction, which can easily lead to poor postoperative reduction and, hence, femoral head necrosis.

In this study, the incidence of postoperative femoral head necrosis in patients with satisfactory fracture reduction and poor reduction was 9.7% (16/165) and 28.2% (24/85), respectively. This finding is similar to that of a study by Wang and Min [5] who reported that the incidence of postoperative femoral head necrosis in patients with poor restoration was 69% (11/16), while the incidence of femoral head necrosis in patients with satisfactory reduction was only 20% (26/130). Femoral head necrosis is mainly caused by poor fracture reduction and, often, the rotation of the femoral head and varus, which results in increased shear force on the fracture surface, affecting the reconstruction of blood vessels around the femoral head and leading to necrosis of the femoral head. In addition, poor restoration, poor matching between the femoral head and acetabulum, and changes in the stress distribution of the femoral head result in stress concentration on the surface of the femoral head and readjustment of the ultrastructure of the internal trabecular bone, which can also cause necrosis of the femoral head.

In related research studies, the role of BMI has not been taken seriously. In this study, the incidence of ONFH was higher in patients with obese BMI after analysis, suggesting that high preoperative BMI may increase the risk of fracture displacement. The literature also suggests that the muscle pulling force around the pelvis is the most important factor affecting local stress at the femoral neck fracture. In addition, BMI can partially reflect the muscle condition of the whole body. For unstable fractures, high BMI may lead to increased fracture stress due to the local muscle strength at the fracture, which increases the incidence of re-displacement, resulting in an increased probability of non-union or necrosis of displaced fractures. In addition, the incidence of hyperlipidemia in obese people is significantly higher than that in non-obese people, and hyperlipidaemia will form fat plugs that prevent the formation of new blood vessels, thereby leading to femoral head necrosis. Therefore, the authors believe that BMI may indirectly influence the healing of femoral neck fractures by influencing parameters such as fracture local stress and blood lipids. Changes in BMI values and blood lipids after follow-up may be important for femoral neck fracture healing and thus require further study. At the same time, our study showed that ASA classification (III, IV) is also one of the factors that should be paid attention to. Complication rates after surgery for hip fractures in adults were associated with pre-existing congestive heart failure and high ASA classification [20].

Few studies have investigated whether internal fixation should be performed after femoral neck fracture surgery and the effect of internal fixation on femoral head necrosis. Chinese scholars Sun suggested that after removal of the internal fixation, the original fracture end will bear all compressive, tensile, and shear stresses, leading to changes in local biostress, partial remodeling of the bone plate, and restoration of the ultrastructure of the trabecular bone. Adjustment may cause vascular malformation occlusion, as when nails are removed, blood flow around the femoral head can be destroyed, stimulating local vasospasm. The blood then exhibits a hypercoagulable state, and the blood supply around the femoral head is reduced. Under these conditions, a local thrombus is easily formed, and surgical operations can lead to intra-articular haematoma and intra-articular pressure increases, further affecting the femoral head blood flow and resulting in femoral head necrosis. In contrast, univariate analysis of this group of patients showed that removal of internal fixation can reduce the rate of postoperative femoral head necrosis, but multivariate analysis showed that internal fixation retention is not an independent risk factor affecting femoral head necrosis. The authors speculate that the presence of internal fixation can cause increased pressure in the femoral head and aggravate the femur.

Head ischemia, which increases the rate of necrosis of the femoral head, and internal fixation removal, which can be equivalent to the decompression of the medullary cavity, are conducive to re-establishing the blood supply to the femoral head. However, all the scholars have explained that the causes of femoral head necrosis are speculative and that there is no experimental support for how to address internal fixation after femoral neck fractures or for its impact on femoral head necrosis.

Whether postoperative weight-bearing time affects the femoral head necrosis rate remains controversial. Huang et al. reported that the rate of femoral head necrosis was significantly higher in patients who walked in less than 3 months after surgery than in those who walked at 3 to 6 months and 6 months after the surgery. There was no significant difference in the femoral head necrosis rate. In the above groups of patients, the rates of bone necrosis after surgery were < 21.5% (5/23), 15.7% (31/197), and 13.3% (4/30), respectively. The difference was not statistically significant, indicating that the rate of bone necrosis after operation was similar among patients who walked at < 3 months, 3–6 months, and > 6 months after surgery. Loading time had no significant effect on the incidence of femoral head necrosis. Whether the time from injury to surgery affects the incidence of postoperative femoral head necrosis is also controversial. Szita et al. reported [21] that the femoral head necrosis rate was 10.5% in the surgical group within 6 h after injury, which was significantly lower than 20% in the surgical group after 6 h postoperatively. Early reduction and effective internal fixation after injury can increase the blood supply to the femoral head, and blood supply reduces the occurrence of femoral head necrosis. Other scholars believe that delayed surgery to 6–36 h after injury does not affect the incidence of postoperative femoral head necrosis. In this group, the incidence of femoral head necrosis was 16.5% (14/85) and 15.8% (26/167), respectively, in patients who underwent surgery at ≤ 24 h and > 24 h after injury. The difference was not statistically significant, suggesting that the time to surgery can be postponed to 24 h after injury without affecting the incidence of femoral head necrosis. Because the blood supply of the femoral head is mainly affected by the momentary detonation force at the time of trauma, it is not related to when the surgery is performed.

This study also has some limitations. First, this study is a retrospective study with inherent and well-known limitations and prejudices. Second, the authors did not have patients with kidney disease, liver disease, and other diseases, which may affect the development of femoral head necrosis. Third, the patient was treated by multiple surgeons. The level of surgeon experience and type of implant may have some impact on complications. A small number of patients limit the ability to make clear statistical conclusions; however, this limitation exists in any institutional study due to the rarity of fractures.

## Conclusion

In summary, a variety of risk factors are involved in the occurrence of femoral head necrosis after treatment of adult femoral neck fractures with hollow compression screws. This study found that removal of internal fixation, type of fracture, quality of reduction, BMI and ASA classification were the most important risk factors influencing the development of femoral head necrosis. And these ratios also increased the occurrence of femoral head necrosis. In this treatment, there are high-risk factors, and targeted measures should be taken to reduce the risk of femoral head necrosis.

## Data Availability

All data generated or analyzed during this study are included in this published article, and the supplementary file. We do not wish to share our patients’ data because it involves patient’s privacy.

## Abbreviations

ONFH: Osteonecrosis of the femoral head
ASA: American Society of Anaesthesiologists
